# Bio-artificial bone formation model with a radial-flow bioreactor for implant therapy—comparison between two cell culture carriers: porous hydroxyapatite and β-tricalcium phosphate beads

**DOI:** 10.1007/s13577-018-0218-x

**Published:** 2018-10-01

**Authors:** Hideki Nomoto, Haruka Maehashi, Misako Shirai, Mariko Nakamura, Takahiro Masaki, Yoshihiro Mezaki, Jonghyuk Park, Mamoru Aizawa, Kiyoshi Ohkawa, Kiyotsugu Yoshida, Tomokazu Matsuura

**Affiliations:** 1 Department of Biochemistry, The Jikei University School of Medicine, Tokyo, Japan; 2 Department of Laboratory Medicine, The Jikei University School of Medicine, 3-25-8 Nishi-shinnbashi, Minato-ku, Tokyo, Japan; 3 Laboratory of Biomaterials, Department of Applied Chemistry, School of Science and Technology, Meiji University, Kawasaki, Japan

**Keywords:** Bio-artificial bone, Bioreactor, β-Tricalcium phosphate (β-TCP), Hydroxyapatite (HA), Implant, Glucose metabolism, ^13^CO_2_ exhaust test, Sulfotransferase (SULT) 1, Vitamin A metabolism

## Abstract

Bone grafting is necessary before dental implant treatment in patients with jaw bone defects. Currently, autologous bone grafting is a major burden on the patient. However, it is impossible to form a sufficient foundation for the implant with a bone-filling agent alone. It is, therefore, necessary to prepare hybrid artificial bone tissue containing osteoblasts and osteoclasts. In this study, mouse MC3T3-E1 pre-osteoblast cells and human embryonic-derived osteoblastic cell line hFOB1.19 were cultured in radial-flow bioreactors (RFB) to form three-dimensional artificial bone filled with porous beads of β-tricalcium phosphate (β-TCP) or hydroxyapatite (HA)—which are clinically used as bone-filling agents—as cell culture carriers. When circulation culturing was performed in the growth medium for the first 10–12 days, glucose consumption was increased in the cultures with HA beads in comparison to the cultures with β-TCP beads. When cultured in the differentiation culture medium during the second half of the culture period, the glucose consumption decreased in the culture with HA beads. A DNA microarray analysis suggested that osteogenesis progressed fast in three-dimensional culture filled with HA beads and that partly differentiation into osteoblasts was prominent in cultures with β-TCP beads. In the growth process of MC3T3-E1 cells, the vitamin A metabolism was also activated, the synthesis and degradation of retinoic acid was enhanced, and the metabolism of the same process decreased at the end of differentiation in three-dimensional cultures. Three-dimensional circulation culture in RFB is considered to be useful for the formation of hybrid bio-artificial bone tissue.

**Electronic supplementary material:**

The online version of this article (10.1007/s13577-018-0218-x) contains supplementary material, which is available to authorized users.

## Introduction

When teeth are lost due to dental caries, periodontal disease, or trauma, the implantation is useful for restoring the chewing function [1]. To perform implant treatment, it is necessary to directly fix an artificial dental root—which is made of a titanium alloy or a similar material—for fixing the artificial tooth to the jawbone [2]. However, when the jaw bone that is to become the foundation is thinned, autologous bone must be transplanted before implantation [3]. An alternative to autogenous bone grafting is the transplantation of artificial bone; at present, ceramics such as porous hydroxyapatite are used [4, 5]. However, in order for artificial bone to engraft, osteoblasts adhere to artificial bone matrix, and bone formation needs to occur to form a foundation for the implant [6]. At present, it is difficult to form strong bone for transplantation procedures using ceramics alone [7].

It is necessary to fabricate artificial bones engrafted with osteoblasts and osteoclasts outside the body and transplant them to repair jawbone defects to form a healthy jawbone. In the present study, a radial-flow bioreactor (RFB) was filled with granules of porous hydroxyapatite (HA) or β-calcium phosphate (β-TCP), followed by three-dimensional perfusion culture of mouse-derived MC3T3-E1 cells and human embryonic-derived osteoblastic cell line hFOB1.19 to attempt induction of differentiation of the cells into bone tissue [8–13]. The results between an ordinary monolayer culture and 3-dimensonal culture were compared using a deoxyribonucleic acid (DNA) microarray.

## Materials and methods

### Cell cultures

The osteoblast-like mouse cell line MC3T3-E1 was maintained in minimum essential medium alpha (α-MEM) medium containing 10% fetal bovine serum (FBS) [11]. For monolayer cultures, the cells were inoculated into 75-cm flasks at a density of 30,000–50,000/cm^2^. From 24 h after the start of culturing, the medium was renewed every third day with α-MEM containing 10% inactivated FBS and supplemented with 2 mM l-ascorbic acid 2-phosphate and 4 mM β-glycerophospahte to induce osteoblastic differentiation.

hFOB1.19 cells were received from ATCC (ATCC^®^ Number: CRL-11372™) temperature-sensitive SV40 large T antigen gene was transfected into this cell, it is expressed at 34 °C and its expression stops at 39 °C. Therefore, in growth culture, 10% of inactivated FBS and 1 g/L of glucose were added to the DMEM/F12 GlutaMax culture solution and cultured at 34 °C. In the differentiation culture, l-ascorbic acid 50 µg/mL, vitamin D 3 × 10^−8^ M, vitamin K 3 × 10^−8^ M was added to the growth medium and incubated at 39 °C. The culture broth was changed almost every 3 days.

In the monolayer culture, gene expression analysis was performed after differentiation culture for 10 days after cell seeding.

### Three-dimensional perfusion culture

Either granules of porous HA (Apaceram, PENTAX-HOYA, Tokyo) or granules of β-TCP (OSferion, Olympus, Tokyo) were used as the cell culture carrier for the 3-dimensional perfusion culture. A 5-mL radial-flow bioreactor (RFB; Able-Biott, Tokyo) was filled with the cell culture carrier, and the culture carrier was inoculated with 7.5 × 10^6^ cells, flowing out of the reservoir. In MC3T3-E1cells, culturing by means of perfusion with 100 mL α-MEM culture medium (circulation speed 1000 mL/h) was continued for 12 days, with half of the medium renewed at intervals of 1–2 days. Culturing was then continued for another 12 days with the medium renewed every 2 days with α-MEM containing 10% inactivated FBS and supplemented with 2 mM l-ascorbic acid 2-phosphate, 1 g/L of glucose and 4 mM β-glycerophosphate.

On the other hand, in hFOB 1.19 cells, 7.5 × 10^6^ cells were seeded in RFB and cultured in 100 mL growth medium at 34 °C for 10 days. Thereafter, it was cultured in a differentiation medium at 39 °C for 14 days.

### Follow-up of three-dimensional culturing by a ^13^C-glucose expired gas test

To confirm the viability of the cells in the RFB over time, 12.5 mg/100 mL of ^13^C-glucose (Chlorella Industry Co., Ltd., Tokyo) was added to the culture medium and the exhaust gas was collected continuously from the reservoir. The ratio of ^12^CO_2_ to ^13^CO_2_ level in the exhaust gas was measured by infrared spectroscopy (POCone, Otsuka Electronics Co., Ltd., Tokyo). The glucose levels in culture medium were measured by a blood glucose test meter (Arkray, Tokyo).

### RNA extraction

DNA microarray analysis was carried out using ribonucleic acid (RNA) extracted from the following cells: (1) monolayer MC3T3-E1 culture–cells maintained in α-MEM containing 10% immobilized FBS, differentiation-induced cells in α-MEM containing 10% immobilized FBS and supplemented with 2 mM l-ascorbic acid 2-phosphate and 4 mM β-glycerophosphate; (2) three-dimensional culture–differentiation-induced cells in OSferion or Apaceram carrier. The total RNA in cells was extracted using Trizol reagent (Ambion) according to the manufacturer’s instructions. The amount of total RNA was measured using a Nano-Drop ND-1000 spectrophotometer.

### DNA array

Four samples of ribonucleic acid (RNA) were placed on the DNA microarray chip (Agilent Technologies, Inc., Tokyo) for the simultaneous analysis of the expression levels of all the relevant genes. Variations in the gene expression levels were determined by comparing the following four types of cells under six settings: (1) cells in monolayer maintenance culture, (2) cells in monolayer differentiation-inducing culture, (3) cells in three-dimensional culture with differentiation-inducing β-TCP, and (4) cells in three-dimensional culture with differentiation-inducing HA. The comparison of the cells in monolayer culture and 3-dimensional culture showed larger differences in the gene expression levels. Based on this result, we set the cutoff level for the gene comparisons in monolayer and three-dimensional cultures to a five- to tenfold difference in the expression level, and a gene ontology enrichment analysis (GO analysis) was carried out at the elevated cutoff level. As a result, statistically significant GO terms were extracted.

### Real-time PCR

The cDNA generated from total RNA by reverse transcription (RT) using oligo (dT)12–18 primers (Invitrogen) and superscript RT (Invitrogen) in an Applied Biosystems 2720 Thermal Cycler (Applied Biosystems). The RT steps consisted of incubation at 65 °C for 5 min followed by incubation at 50 °C for 60 min and 75 °C for 15 min. The RT products were used as templates for the PCR amplification of 20-µL reactions. A real-time polymerase chain reaction (PCR) was performed with 10 µL of SYBR Green Mix, 0.4 µL of each primer, 2 µL of cDNA template, and 7.2 µL of dH_2_O. The mixture was incubated at 95 °C for 10 s and 60 °C for 1 min for 40 cycles, terminating at 95 °C for 15 s, 60 °C for 1 min, and 95 °C for 15 s. Changes in the expression levels of alcohol dehydrogenase (ADH) 1, aldehyde dehydrogenase (ALDH) A1 and SLUT1 were examined using a StepOnePlus real-time PCR system (Thermo Fisher Scientific KK, Yokohama).

## Results

### The assessment of cell viability by the ^13^C-glucose expired gas test

**Fig. 1:**
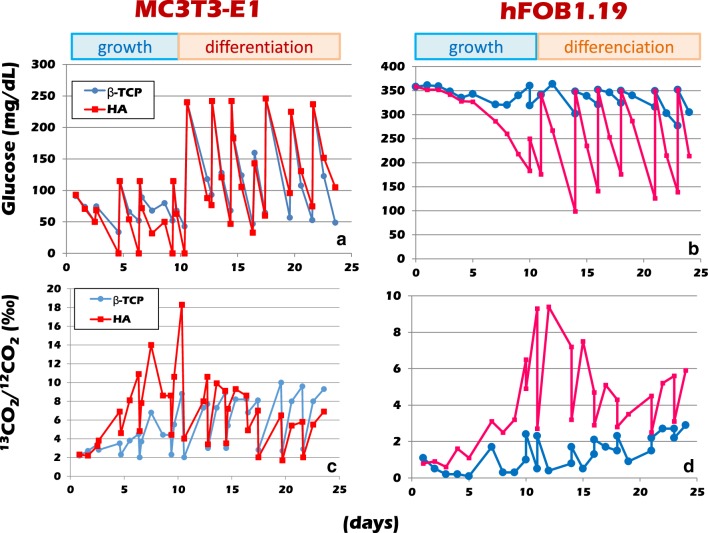
The progress of RFB circulation culture: MC3T3-E1 cells were seeded in RFB filled with porous β-TCP or HA beads, and cultured under circulation with a growth medium for the first 12 days and a differentiation medium for the second 12-day period. ^13^C-glucose was added in both media. In the growth culture medium, glucose consumption was active in the cells attached to the HA beads. In contrast, when circulation culture was performed with a differentiation medium containing high glucose, the glucose consumption was more gradually increased in the RFB filled with β-TCP beads than in HA beads, and more ^13^ CO _2_ was discharged from the RFB filled with HA beads than from the RFB filled with β-TCP beads

When three-dimensional culturing was carried out using β-TCP as the carrier, the ^13^CO_2_ expiration was relatively constant in both the proliferation culture medium and the differentiation culture medium. In MC3T3-E1 cell culture carried out using HA as the culture carrier, a higher level of ^13^CO_2_ expiration was seen in the proliferation culture medium, indicating active proliferation. After the medium was switched to the differentiation culture medium, the ^13^CO_2_ formation decreased to a level comparable to that seen in the culture carried out using β-TCP (Fig. 1a). The course of oxygen consumption also reflects the level of ^13^CO_2_ expiration, and the glucose consumption of MC3T3-E1 cells cultured on HA beads increased in the growth medium in comparison to that in MC3T3-E1 cells cultured on β-TCP beads. In contrast, it decreased in the differentiation culture (Fig. 1b).

Even in hFOB 1.19 cells, when HA was used as a culture carrier, glucose consumption increased markedly by circulation of the differentiation medium (Fig. 1c). In addition, the production of ^13^CO_2_ also increased (Fig. 1d). On the other hand, when β-TCP was used as a cell culture carrier, glucose consumption. ^13^CO_2_ production remained low in hFOB 1.19 cells even after exchange to differentiation medium, and both glucose consumption and ^13^CO_2_ production tended to increase after 10 days.

### GO analysis

GO analysis of MC3T3-E1 cells were performed, when β-TCP and HA are used as culture carriers. The genetic group related to “development” and “differentiation” increased and the “immune response” decreased in β-TCP carriers. The genes related “extracellular region” increased with “development” in HA carriers.

The results of GO analysis of hFOB 1.19 cells are shown in Table 1. Comparison was made between cells cultured in RFB filled with HA or β-TCP carriers in differentiated condition and monolayer culture cells with differentiation medium. When hFOB1.19 cells were cultured in HA carriers, genes related to “intercellular communication” and “extracellular matrix formation” highly expressed. On the other hand, when β-TCP was used as a cell culture carrier, the gene group related to histone H4-K20 demethylation was upregulated.

**Table 1:**
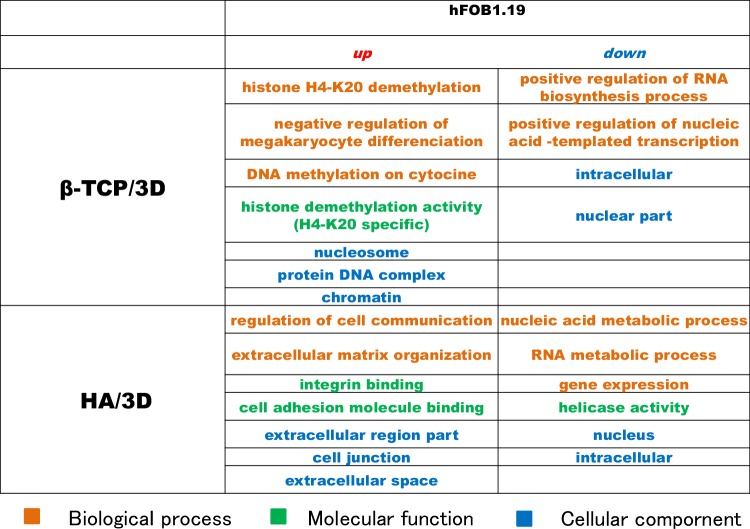
Gene ontology analysis of hFOB 1.19 cells

### Pathway analysis

In the pathway analysis, a comparison between the MC3T3-E1 cells in monolayer differentiation culture and the cells in three-dimensional culture carried out using β-TCP as the differentiation-inducing agent showed an increase in the expression levels of ecto-nucleotide pyrophosphatase/phosphodiesterase (ENPP1), Collagen 10A1, vascular endothelial growth factor (VEGF) and matrix metalloproteinase 13 (MMP13), which are known to be expressed at the stage of osteocyte invasion after extracellular matrix (ECM) dissolution. In contrast, the expression level of insulin-like growth factor-1 (IGF1), which is known to increase at the time of chondrocyte proliferation, was decreased (Supplementary data 1). A comparison between the cells in monolayer differentiation culture and those in three-dimensional culture performed using HA as the differentiation-inducing agent revealed relative increases in the expression levels of ENPP1, Collagen 10A1 and MMP13, but no evidence of VEGF induction. A comparison between the monolayer culture cells and differentiation-inducing culture cells suggested differentiation towards ossification in the cells in three-dimensional culture with β-TCP or HA (Supplementary 2). A comparison between the cultures with β-TCP and HA as the differentiation-inducing agent revealed a relative increase in the expression levels of Adamts5, MMP9 and MMP13 (more differentiation towards ossification) in the cells cultured with HA than in the cells cultured with β-TCP. The expression levels of the genes encoding IGF1 and IGF2 were higher in the HA cultures, suggesting that the cellular proliferation was more active in the cultures carried out using HA. The expression level of fibroblast growth factor 18 (FGF18), known to be expressed during cartilage hypertrophy, was relatively low in the HA cultures, while the expression level of intraflagellar transport protein 88 (IFT88) was high in the same cultures; thus, there was a discrepancy between these two genes. With regard to the expression levels of MMP, the expression levels of MMP8, MMP13, MMP9 and MMP3 tended to be higher in the HA cultures than in the β-TCP cultures.

The induction of differentiation in monolayer culture revealed increased expression levels of genes related to the “extracellular region”. Switching the monolayer culture to three-dimensional culture resulted in the reinforcement of the genes classified as “development” genes. Culturing with HA resulted in greater reinforcement of the “extracellular region” genes in comparison to all other types of culture. Culturing with β-TCP resulted in the reinforcement of genes related to “development” or “differentiation”. In the pathway analysis of MC3T3-E1 cells, a comparison among the groups led to the extraction of “matrix metalloproteinases,” “focal adhesion,” and “endochondral ossification,” as duplicated pathways.

In the gene expression pathway analysis of hFOB 1.19 cells cultured on HA carriers, the upregulated pathways of “glycolysis and glyconeogenesis”, “vitamin D receptor pathway”, and “matrix metalloproteinases” were higher ranked compared to monolayer differentiation culture (Table 2). For example, with regard to the glycolysis system, the expression of hexokinase 2, a rate-limiting enzyme, increased 10.5-fold in HA carrier culture compared to monolayer differentiation culture (Supplementary 3). On the other hand, it was 1.91 times in the β-TCP culture, which well reflected the increase in glucose consumption in HA. Meanwhile, Histon modifications were ranked at the top of pathway in β-TCP culture, which was the result of supporting the results of GO analysis.

**Table 2:** Pathway analysis of hFOB 1.19 cells cultured in HA carriers

Pathway (upregulated)	*n*	*r*	*z*	*p*
Glycolysis and gluconeogenesis	18	9	4.11	0.000
Vitamin D receptor pathway	117	32	3.68	0.000
Ganglio sphingolipid metabolism	15	7	3.39	0.001
Photodynamic therapy-induced NF-kB survival signaling	30	11	3.28	0.003
Photodynamic therapy-induced HIF-1 survival signaling	34	12	3.27	0.004
Lung fibrosis	43	14	3.17	0.001
miRNA targets in ECM and membrane receptors	20	8	3.09	0.001
Matrix metalloproteinases	20	8	3.09	0.005
Inflammatory response pathway	17	7	2.98	0.009
Osteoclast signaling	14	6	2.88	0.007

Pathway (down-regulated)	*n*	*r*	*z*	*p*
Insulin signalling in human adipocytes (normal condition)	7	4	4.08	0.002
Insulin signalling in human adipocytes (diabetic condition)	7	4	4.08	0.001
RIG-I-like Receptor signaling	52	14	3.95	0.000
RNA interference	5	3	3.65	0.003
Transcription factor regulation in adipogenesis	16	6	3.58	0.003
Hypothesized pathways in pathogenesis of cardiovascular disease	23	7	3.18	0.004
Lidocaine metabolism	1	1	2.95	0.009
Gastric cancer network 1	25	7	2.91	0.009
mRNA processing	124	22	2.75	0.004
Canonical and non-canonical TGF-B signaling	17	5	2.59	0.014

*n* number of genes to be analyzed in pathway, *r* number of genes extracted by fold change (2×), *z* threshold of fold change, *p* permuted *p* value

### On osteoblast differentiation

**Fig. 2:**
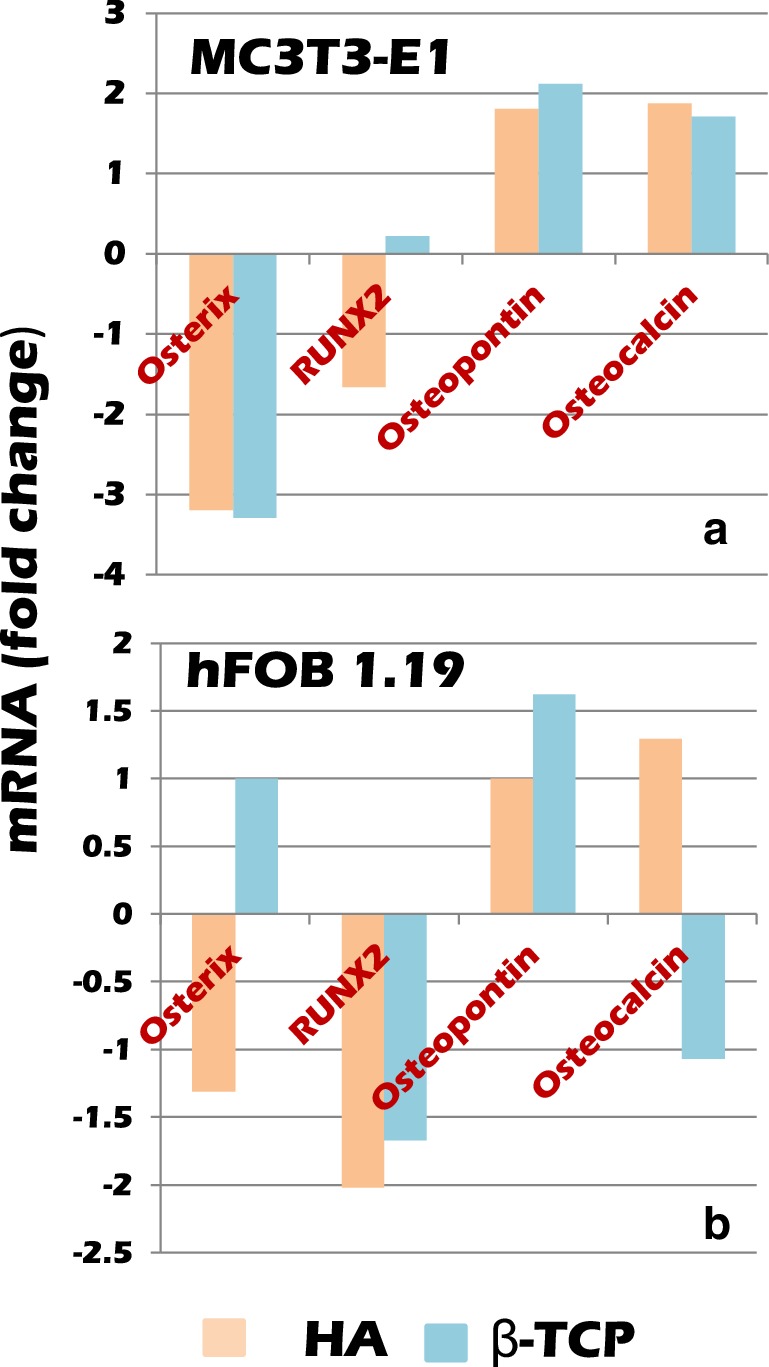
MC3T3-E1 cells and hFOB 1.19 cells were cultured under circulation in a radial-flow bioreactor for about 3–4 weeks (about 2 weeks after changing to differentiation medium). mRNA Expression of Osterix, RUNX 2 as pre-osteoblast markers, and expression of osteopontin and osteocalcin as osteoblast markers were compared. HA or β-TCP carriers were packed in the bioreactors. MC3T3-E1 cells appeared to be differentiated into osteoblasts almost during this period regardless of which carrier they were cultured. On the other hand, h-FOB 1.19 cells seemed to differentiate into osteoblasts in HA carriers, but it seemed that the differentiation to osteoblasts did not proceed sufficiently in β-TCP carriers until this period

In gene expression related to osteoblast differentiation of hFOB 19.9 cells, expressions of Osterix and RUNX 2,which were markers of pre-osteoblast, were lower in HA culture than in monolayer differentiation culture, and expression of osteopontin and osteocalcin, which were markers of osteoblast, tended to be upregulated. In β-TCP carriers, RUNX 2 was suppressed, and osteopontin was upregulated, but Osteocalcin did not. There is a possibility that they may be differentiated to osteoblasts in HA carriers at this time. On the other hand, it was suggested that β-TCP culture has not yet reached the differentiation to osteoblasts (Fig. 2; Table 3).

**Table 3:** Pathway analysis of hFOB 1.19 cells cultured in β-TCP carriers

Pathway (upregulated)	*n*	*r*	*z*	*p*
Histone modifications	61	13	4.48	0.000
NAD biosynthesis II (from tryptophan)	7	3	3.76	0.001
Nicotine activity on chromaffin cells	1	1	3.68	0.005
Diclofenac metabolic pathway	1	1	3.68	0.007
Gastric acid production	1	1	3.68	0.003
ID signaling pathway	12	4	3.62	0.005
FTO obesity variant mechanism	4	2	3.41	0.006
miR-222 in exercise-induced cardiac growth	4	2	3.41	0.014
Pyrimidine metabolism	13	4	3.41	0.001
Eicosanoid synthesis	14	4	3.21	0.004

Pathway (down-regulated)	*n*	*r*	*z*	*p*
GPCRs, Class C metabotropic glutamate, pheromone	7	3	3.88	0.002
NAD+ metabolism	14	4	3.32	0.004
Leptin Insulin Overlap	14	4	3.32	0.002
Ectoderm differentiation	103	15	3.30	0.001
Nucleotide GPCRs	5	2	3.02	0.007
NAD metabolism, sirtuins and aging	10	3	2.99	0.008
Trans-sulfuration pathway	10	3	2.99	0.011
miRNAs involved in DNA damage response	17	4	2.82	0.006
Ovarian infertility genes	17	4	2.82	0.007
Cannabinoid receptor signaling	6	2	2.65	0.014

*n* number of genes to be analyzed in pathway, *r* number of genes extracted by fold change (2×), *z* threshold of fold change, *p* permuted *p* value

### Retinol metabolism

**Fig. 3:**
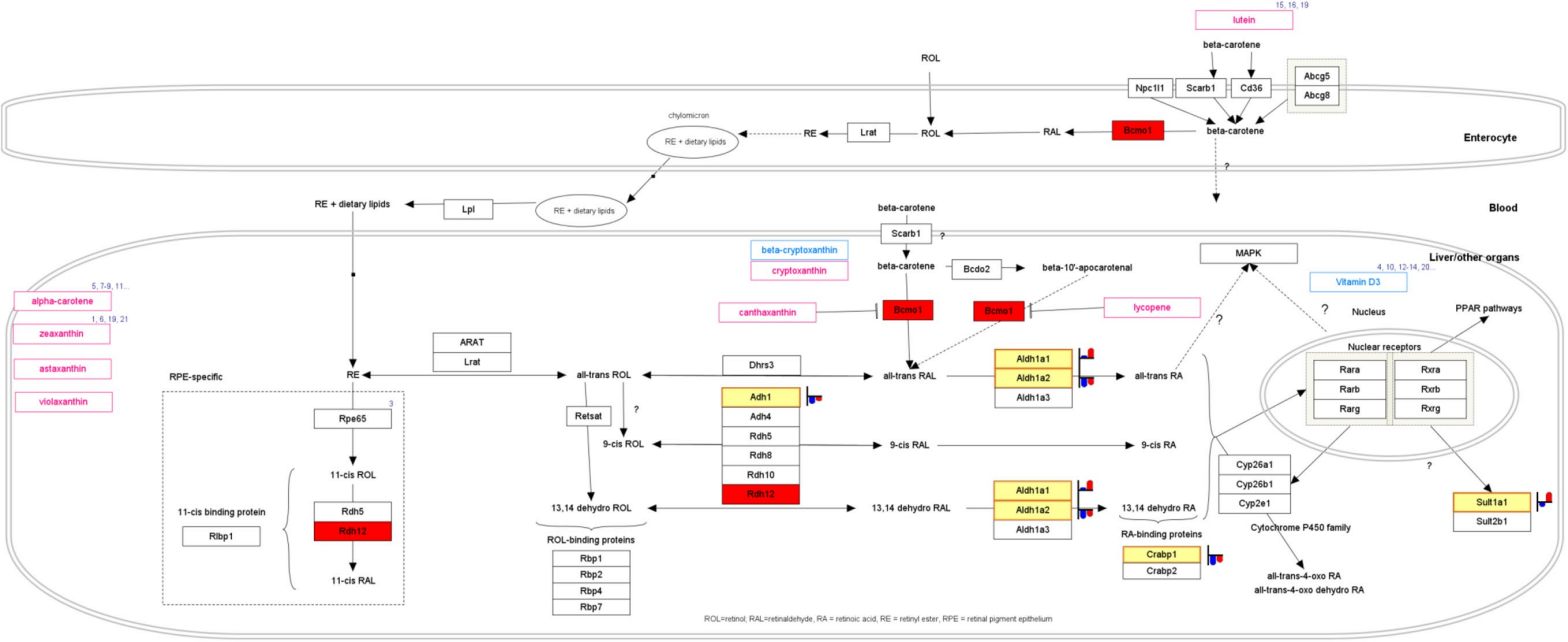
The retinoid metabolism-related gene expression in monolayer-cultured MC3T3-E1 cells in growth medium and in differentiation medium: The retinoic acid production was enhanced when cells were cultured in differentiation medium. Furthermore, since the expression of SULT1 was enhanced, it is presumed that the metabolism of factors involved in transcription (i.e., retinoic acid, vitamin D, steroids) was enhanced

When “retinol metabolism” in MC3T3-E1 cells was analyzed in detail, it was found that among the enzymes known to be involved in hydroxylation of retinol into retinal, alcohol dehydrogenase 1 (ADH1) was induced during monolayer culturing and that the induction of aldehyde dehydrogenase 1a1 (ALDH1A1) and ALDH1A2 (which are involved in the oxidation of retinal into all-trans retinoic acid) occurred. In other words, there was a tendency for the formation of endogenous all-trans retinoic acid (ATRA) through the induction of differentiation into osteoblasts. Actually, there was also a tendency for the induction of cellular retinoic acid-binding protein 1 (CRABP1). As a result, the powerful induction of the SULT1 gene was observed (Fig. 3). On the other hand, a comparison between the cells in monolayer differentiation-inducing culture and those in three-dimensional culture with β-TCP differentiation-inducing agent revealed a reduction in the expression levels of ADH1 and ALDH1A1, with no significant difference in the expression levels of CRABP1 and SULT1. Thus, it seems likely that the formation of endogenous ATRA is induced during the induction of differentiation in monolayer culture, leading to the induction of SULT1, but that during three-dimensional culture with β-TCP, the expression of SULT1 by ATRA is suppressed (Supplementary 4).

**Fig. 4:**
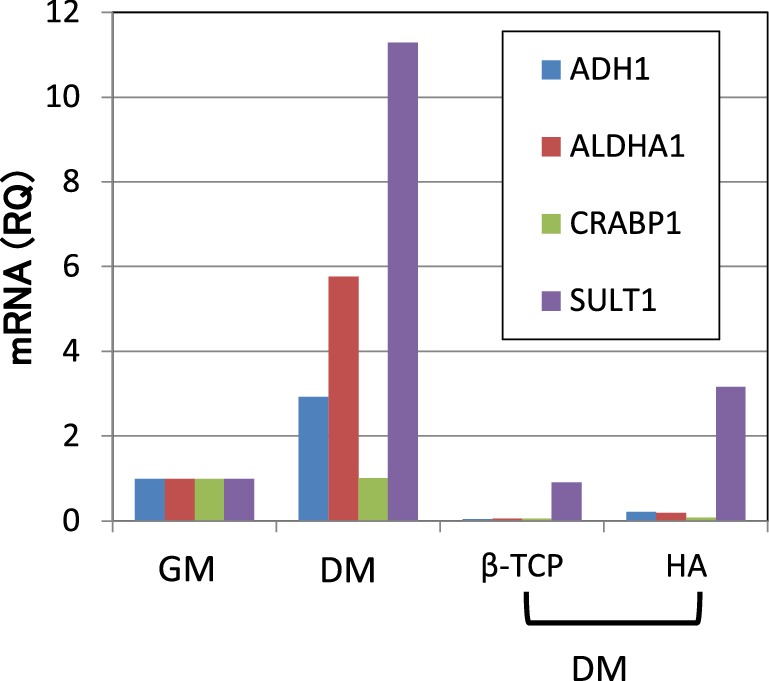
The mRNA expression of enzymes and the binding protein-related retinoid metabolism under each condition: As suggested by the DNA array analysis, in monolayer culture, when cells were cultured in differentiation medium, the metabolism of retinoic acid was enhanced. In contrast, the retinoic acid metabolism was reduced in three-dimensional cultures with either β-TCP or HA beads until the end of three-dimensional culturing. It was suggested that the enhancement of the metabolic system in ossification had converged. *GM* growth medium, *DM* differentiation medium

The confirmation of the same RNA sample by a real-time polymerase chain reaction (PCR) revealed that the induction of differentiation in monolayer culture resulted in the induction of ADH1 and ALDHA1, accompanied by the evident induction of SULT1. In contrast, during three-dimensional culturing with β-TCP or HA, the expression of ADH1 and ALDHA1 was suppressed despite the presence of differentiation-inducing agents; this accompanied by the suppression of the SULT1 expression. The suppression of SULT1 was particularly marked in the culture that was carried out using β-TCP (Fig. 4).

## Discussion

When prosthetic bone is prepared as the base for implant therapy (as a substitute for natural bone), it is ideal that three-dimensional carriers of sufficient strength (e.g., HA and β-TCP) cultured with osteoblasts are prepared for transplantation as an ossified hybrid material (i.e., “viable prosthetic bone”) [14, 15]. Thus, there is a significant technical need of the development for preparing satisfactory prosthetic bone in an extracorporeal setting using a three-dimensional culture method [9, 16, 17]. In the present study, we attempted to prepare prosthetic bone extracorporeally by culturing mouse MC3T3-E1 cells (as a substitute for osteoblasts) in a radial-flow bioreactor with porous β-TCP or HA beads, which are clinically used for transplantation [18–20].

MC3T3-E1 cells were cultured under circulation in culture medium supplemented with ^13^C-glucose in a RFB filled with porous β-TCP or HA beads, and the ^13^CO_2_ exhaust was measured with an infrared spectrophotometer. For the first 12 days, the growth culture medium was supplied, and the second half of the cells in the RFB were cultured in differentiation culture medium. The rate of glucose consumption by the MC3T3-E1 cells cultured with HA beads in the growth medium was high, and they discharged an equivalent amount of ^13^CO_2_. In contrast, when cultured in the differentiation culture solution, the ^13^CO_2_ exhaust decreased. When MC3T3-E1 cells were cultured with β-TCP beads, there was no marked change in the ^13^CO_2_ exhaust, even in the same culture. The mRNA expression of glucose transporter 1 (GLUT1) in three-dimensional culture was approximately twofold that in two-dimensional culture, but there was no significant difference between β-TCP and HA beads. In the latter part of the culture, the glucose consumption and ^13^CO_2_ emissions of the cells cultured with hydroxyapatite beads were lower in comparison to the cells cultured with β-TCP beads, and it was inferred that the state of differentiation was stable. Glucose is indispensable for the differentiation of osteoblasts and it has been proved that a large amount of glucose was consumed by RFBs in three-dimensional culture [21]. Bone is also involved in the regulation of the systemic glucose metabolism and we were of the opinion that the process of the uptake and consumption of a large amount of glucose through GLUT1 during bone formation and the differentiation of osteoblasts could be reproduced in this model [22–24].

We simultaneously carried out a DNA microarray analysis of all of the relevant genes to examine whether or not the cultured cells would differentiate into bone tissue. In this study, both the cells cultured with HA and the cells cultured with β-TCP were found to differentiate into bone tissue. When MC3T3-E1 cells were attached to HA beads and cultured under circulation with differentiation culture medium, extracellular matrix metabolism-related genes were induced, which suggested bone formation. In contrast, when cells were attached to β-TCP beads, genes related to osteoblast differentiation were induced. However, with either bead, the formation of bone and differentiation of osteoblasts ultimately proceeded, suggesting that ossification was advanced. β-TCP is reported to promote the proliferation of osteoblasts, while HA is reported to promote the differentiation of osteoblasts; thus, the authors are of the opinion that results of the present study do not contradict those of other studies [25, 26].

In the β-TCP carriers with differentiation medium, the histone H4-K20 demethylation was nominated to the top rank pathway. In general, methylation of Lys 20 on histone H4 (H4-K20) plays a role in deactivating gene transcription [27]. It was suggested that hFOB 19.9 cells cultured in β-TCP carrier slowly proceeded to proliferation/osteoblast differentiation while suppressing gene expression at about 1 month of culture initiation and 2 weeks of differentiation culture [28], On the other hand, cells cultured in the HA carrier proliferated and differentiated quickly into osteoblasts, and expressed various matrix metalloproteases and start remodeling on HA.

Among the other findings of the present study on MC3T3-E1 cells, based on the reduced expression of SULT1, and more marked differentiation into bone tissue, the state of the cells in the differentiation-inducing culture (which lasted for approximately 2 weeks longer than the cells in monoculture) seemed to be more stable. The results of this study indicate that endogenous ATRA formation is induced during the induction of osteoblast differentiation from monolayer culture, leading to the induction of SULT1 [29, 30]. Sulfotransferases (SULTs) are sulfate conjugate enzymes that account for approximately 15% of the phase II enzymes involved in drug degradation [31]. SULTs are widely expressed in the liver and in hormonally responsive extrahepatic tissues, and are considered to also play a significant role in bone metabolism [32, 33]. SULT1 is inactivated by sulfate conjugation with estrone or estradiol [34, 35]. It suppresses excessive bone turnover through the inactivation of excessive endogenous thyroid hormone. During three-dimensional culturing with β-TCP, the induction of SULT1 by ATRA was reduced, probably indicating the completion of ossification.

In conclusion, we partly succeeded in preparing viable prosthetic bone by culturing osteoblasts in an RFB using porous β-TCP or HA beads (materials clinically used as substitutes for bone) as a cell culture carrier.

In bone remodeling, the osteogenesis period when osteoblasts reconstruct bone tissue is said to be 2–4 months. For this reason, bone formation was incomplete at about 1 month of circulation in RFB, and it became clear that especially when β-TCP is used as a carrier, it tends to slowly differentiate. β-TCP may be suitable for promoting remodeling over time while differentiating into osteoblasts and forming bones with good bone quality.

On the other hand, in the differentiation culture of MC3T3-E1 cells, the induction of SLUT1 which promotes the vitamin A metabolic pathway and the degradation of steroid, estrogen, thyroid hormone, and vitamin D were confirmed. In monolayer culture, osteogenic differentiation seems to proceed relatively quickly, and the increased expression of SLUT1 via RA seems to suggest that the differentiation is halted. In this three-dimensional culture, the osteogenic differentiation is still insufficient, so it is possible that the enhancement of vitamin A metabolism was not observed even in hFOB 1.19 cells, in particular. From now on, to observe up to bone formation, we believe it is necessary to try long-term culture for about 3 months using a bioreactor.

In the future, we will attempt to transplant viable prosthetic bone prepared according to these methods into bone defects and evaluate its potential application as a base for implant therapy and as a bone substitute for filling bone defects.

## Electronic supplementary material

Below is the link to the electronic supplementary material.

Supplementary 1. The ossification gene expression pathway of MC3T3-E1 cells monolayer culture with differentiation medium and three-dimensional culture in an RFB filled with β-TCP beads: MC3T3 E1 cells cultured in an RFB filled with β-TCP beads differentiated into bone cells. Supplementary 2. The ossification gene expression pathway of MC3T3-E1 cells in monolayer culture with differentiation medium and three-dimensional culture in an RFB filled with HA beads: The expression levels of genes involved in ossification were relatively elevated. However, the expression levels of genes involved in cartilage proliferation (i.e., IGF1 and VEGF) did not change. Supplementary 3. Glucose metabolism of hFOB1.19 cells was enhanced in glycolysis system when cultured with HA as a carrier. Expression of hexokinase 2 increased 10.5 times in monolayer differentiation culture and 5.46 times in glucokinase. When β-TCP was used as a carrier, they were 1.91 and 2.33, respectively. Supplementary 4. The retinoid metabolism-related gene expression in monolayer-cultured MC3T3-E1 cells in differentiation medium and three-dimensionally cultured cells in an RFB filled with β-TCP beads: The retinoic acid production was suppressed in three-dimensionally cultured MC3T3-E1 cells in differentiation medium (PDF 1173 KB)
