# Supplementary material for: Bio-artificial bone formation model with a radial-flow bioreactor for implant therapy—comparison between two cell culture carriers: porous hydroxyapatite and β-tricalcium phosphate beads
Source: Hum Cell. 2018 Oct 1;32(1):1–11. doi: 10.1007/s13577-018-0218-x (PMC6315002; doi:10.1007/s13577-018-0218-x)
Supplement: Supplementary file 1 — Supplementary 1. The ossification gene expression pathway of MC3T3-E1 cells monolayer culture with differentiation medium and three-dimensional culture in an RFB filled with β-TCP beads: MC3T3 E1 cells cultured in an RFB filled with β-TCP beads differentiated into bone cells. Supplementary 2. The ossification gene expression pathway of MC3T3-E1 cells in monolayer culture with differentiation medium and three-dimensional culture in an RFB filled with HA beads: The expression levels of genes involved in ossification were relatively elevated. However, the expression levels of genes involved in cartilage proliferation (i.e., IGF1 and VEGF) did not change. Supplementary 3. Glucose metabolism of hFOB1.19 cells was enhanced in glycolysis system when cultured with HA as a carrier. Expression of hexokinase 2 increased 10.5 times in monolayer differentiation culture and 5.46 times in glucokinase. When β-TCP was used as a carrier, they were 1.91 and 2.33, respectively. Supplementary 4. The retinoid metabolism-related gene expression in monolayer-cultured MC3T3-E1 cells in differentiation medium and three-dimensionally cultured cells in an RFB filled with β-TCP beads: The retinoic acid production was suppressed in three-dimensionally cultured MC3T3-E1 cells in differentiation medium (PDF 1173 KB) [file 13577_2018_218_MOESM1_ESM.pdf]

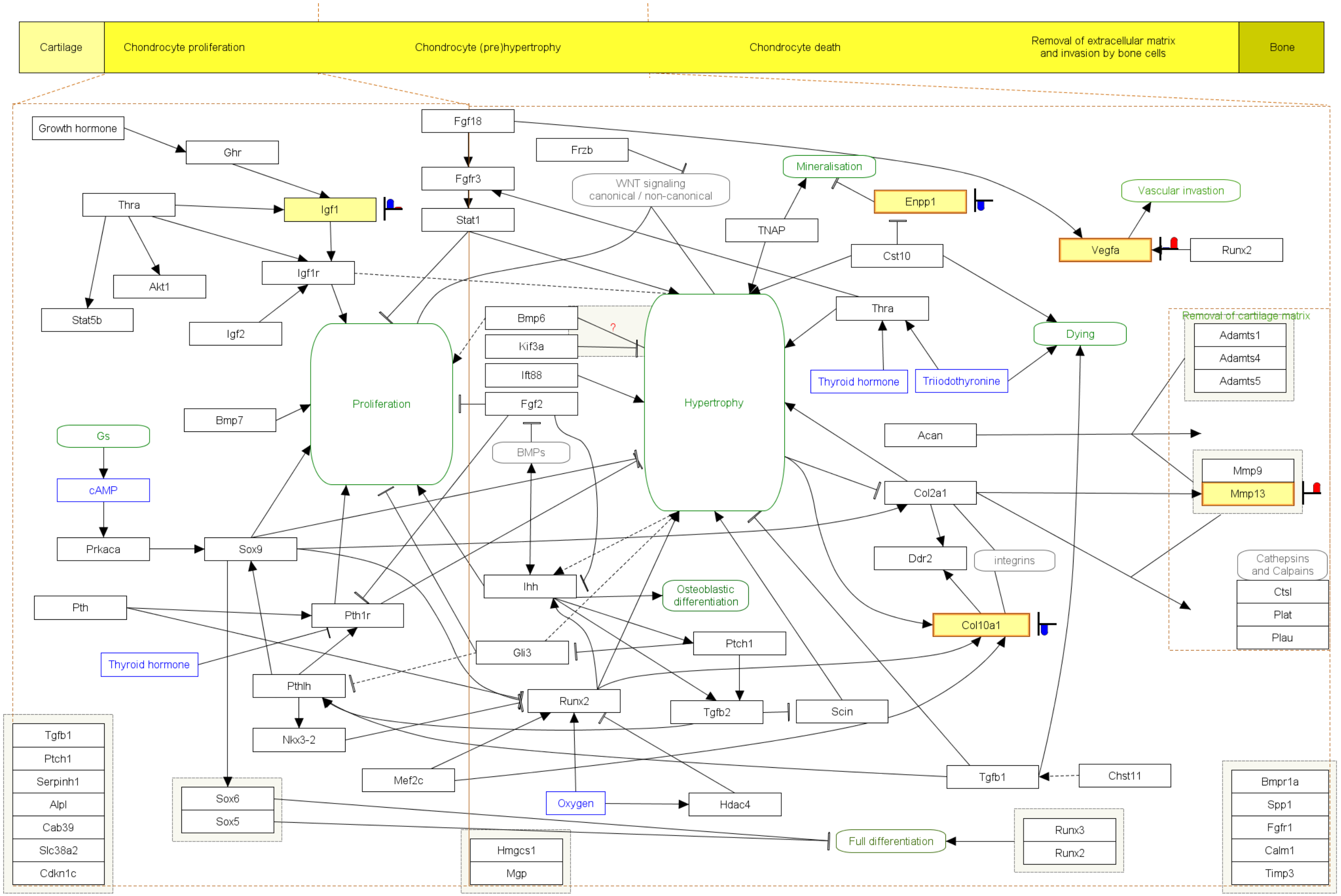

Supplementary 1

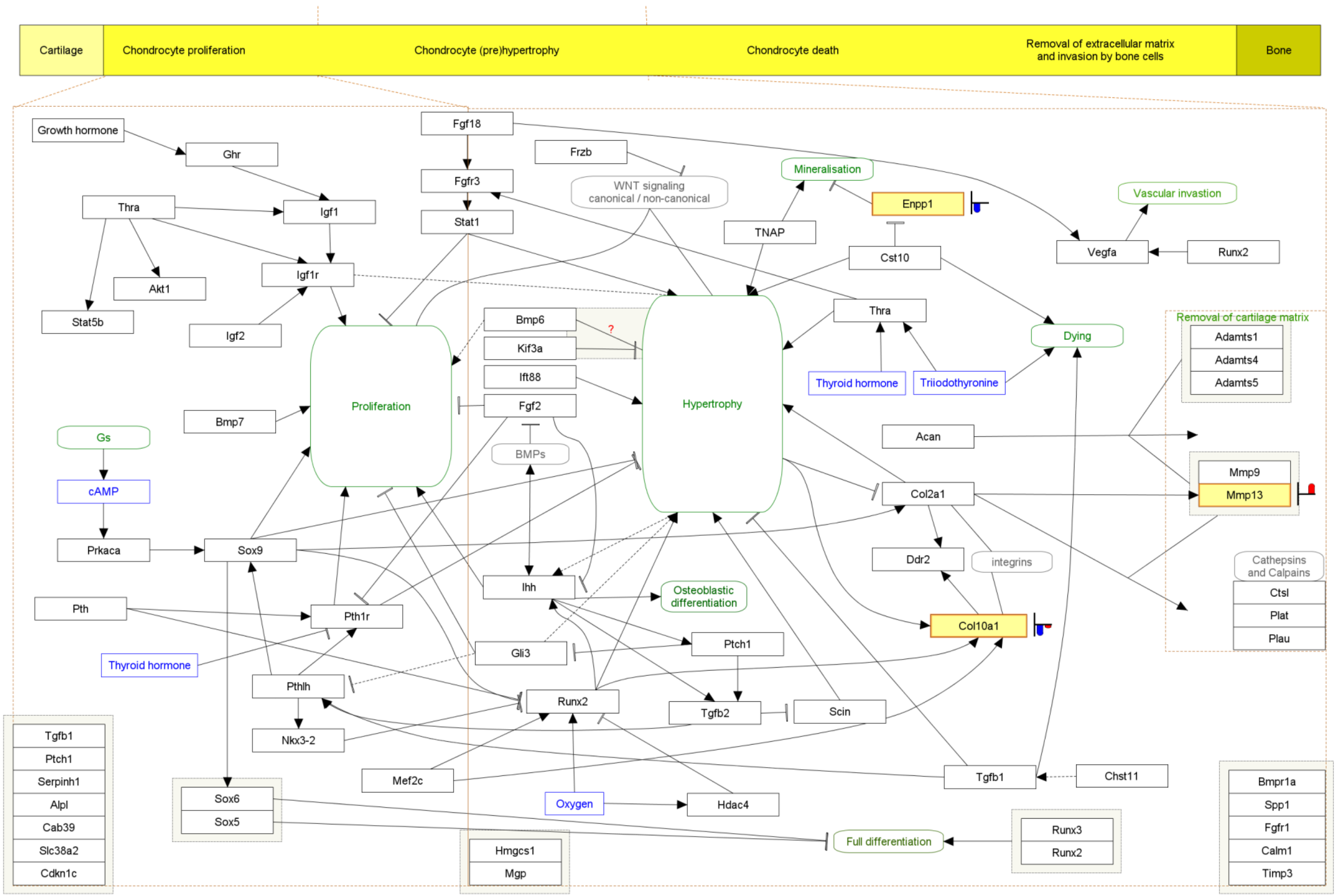

Supplementary 2

Differentiation (HA/3D) vs Differentiation (2D)

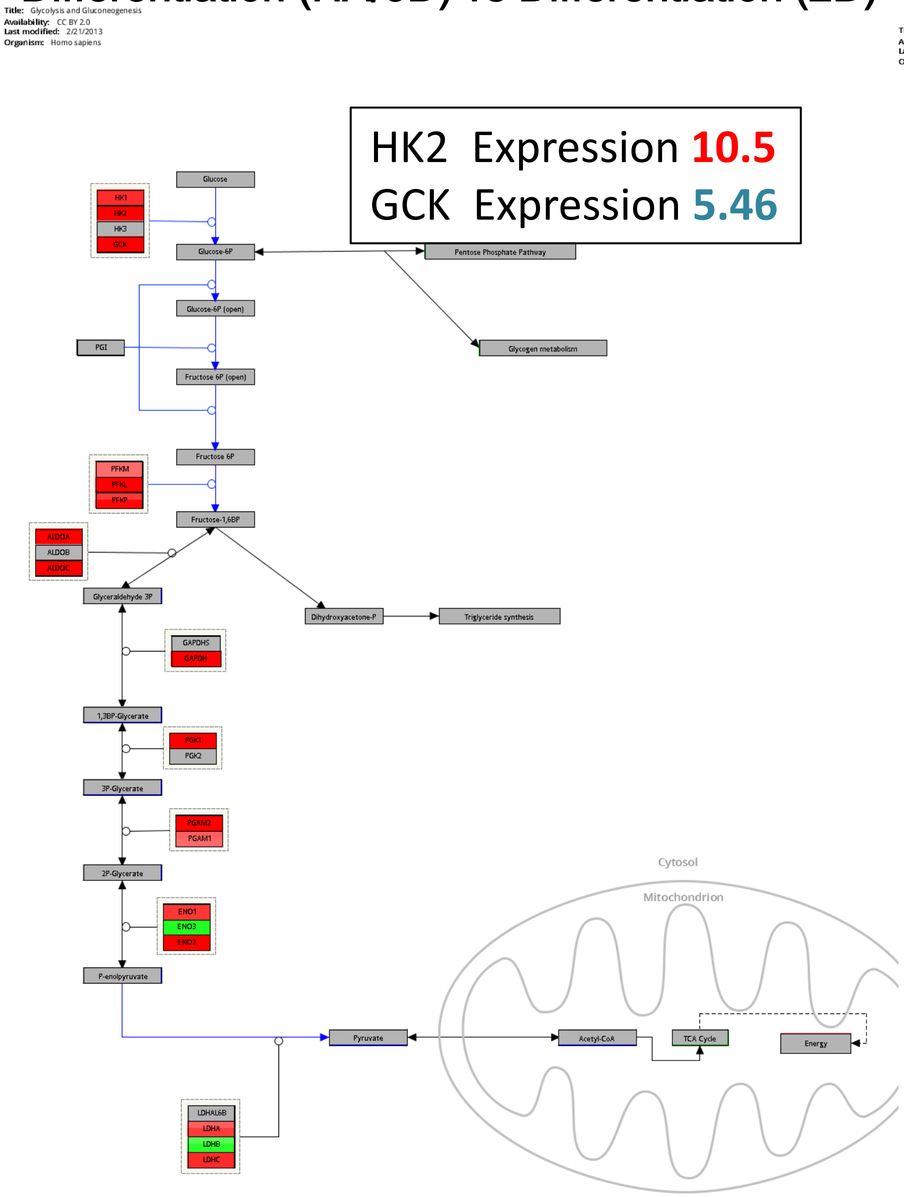

Differentiation (β-TCP/3D) vs Differentiation (2D)

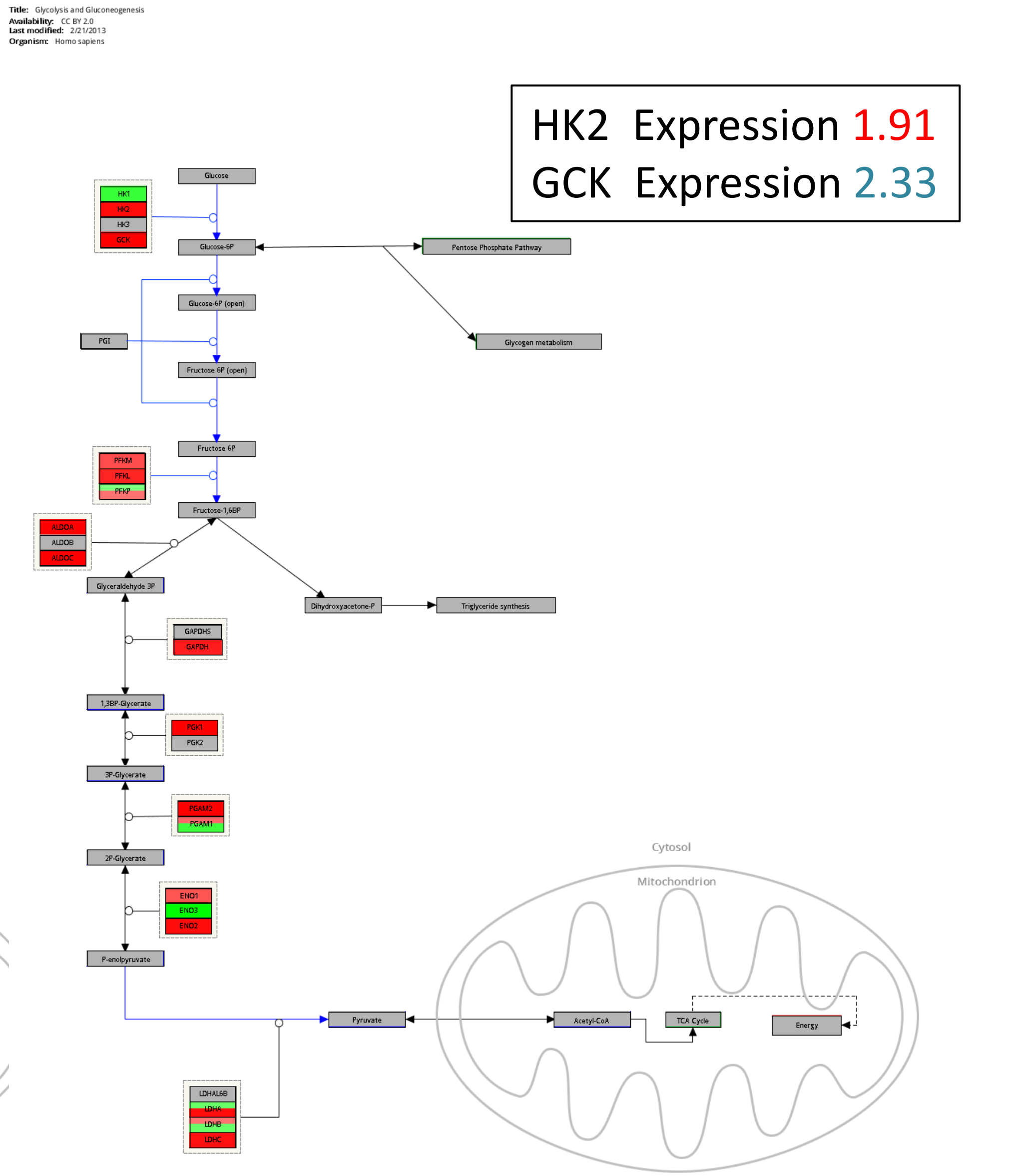

Glycolisis and Glyconeogenesis

Supplementary 3

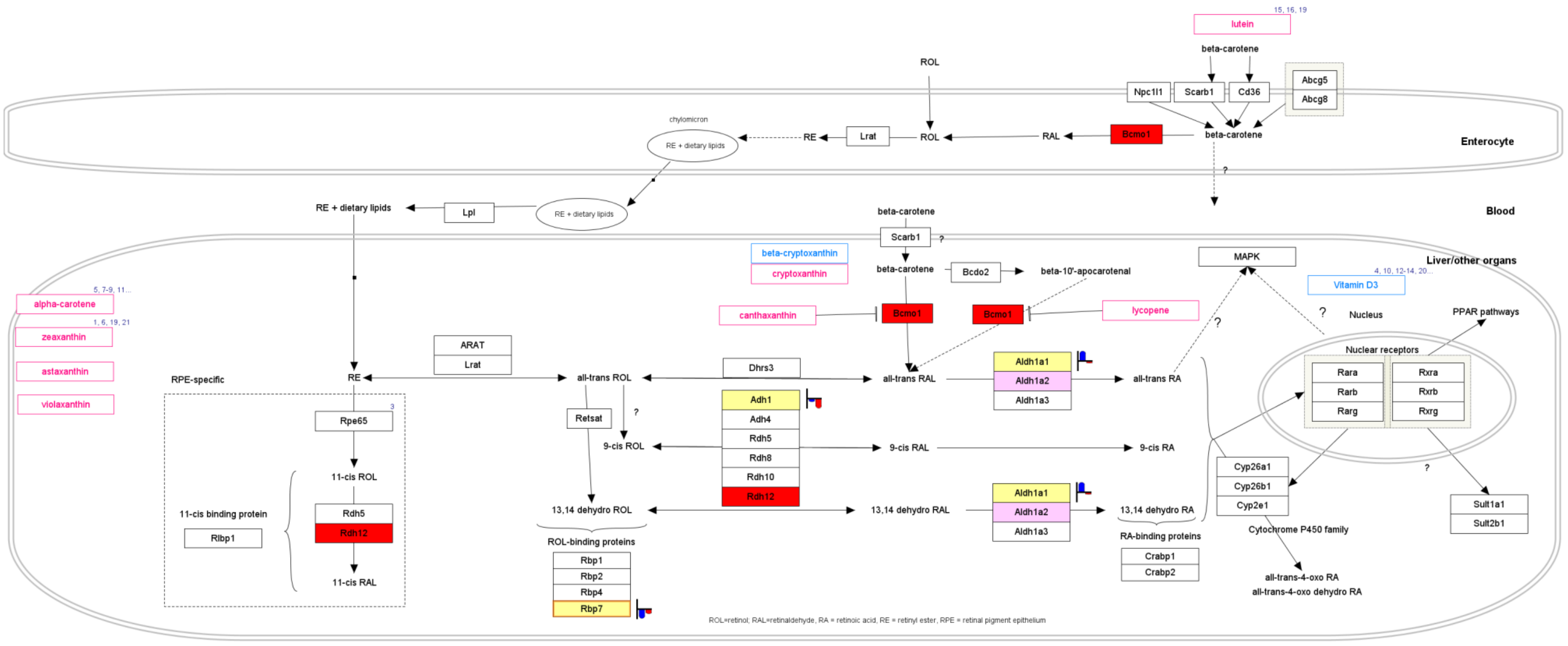

Supplementary 4
